# Structural insights into state transition and end-processing in non-homologous end joining from cryo-EM

**DOI:** 10.1063/4.0001020

**Published:** 2025-10-27

**Authors:** Alex Vogt, Yuan He

**Affiliations:** 1 Northwestern University, Evanston, IL, USA; 2 Johns Hopkins University, Baltimore, MD, USA

## Abstract

Non-homologous end joining (NHEJ) is the major DNA repair pathway used by nondividing cells to mend double-stranded DNA breaks (DSBs). The large protein-DNA complexes that carry out repair have been targets of structural and biophysical investigations which have led to a model in which both DNA ends are held proximate to one another in a protective 'long-range' synaptic state before transitioning to a ligation-competent 'short-range' synaptic state. However, the precise mechanisms that govern how this handoff is achieved remain obscure. Furthermore, there have been inconsistent results in studies investigating the requirements for achieving long-range synapsis, and what role the phosphorylation of one of NHEJ's key factors, DNA-PKcs, plays. Finally, there are a number of related end-processing enzymes that perform critical roles in chemically preparing DNA termini for ligation, but whose interaction with the core NHEJ protein architecture has not been resolved. To address all of these questions, we reconstitute various NHEJ complexes *in vitro* followed by investigation with negative stain and cryogenic electron microscopy paired with orthogonal biochemical and biophysical techniques. With this approach we have identified a key intermediate in the hand-off between long and short range synapsis that contains a single phosphorylated copy of DNA-PKcs, indicating for the first time a mechanism of serial eviction of the two copies of the factor that participate in repair. We have also captured two novel dimeric forms of the DNA-PK complex (DNA-PKcs, Ku70/80, and DNA), both in held together by a relatively obscure scaffolding factor, PAXX. Finally, we report the structure of a short-range synaptic complex in the presence of the NHEJ specific DNA polymerase lambda, which yields new information regarding the myriad end- processing events required for NHEJ are structurally accommodated. In addition to biological insights, this investigation highlights the ability of different EM techniques to capture relatively flexible and less-stable intermediate states by exploring reconstitution and sample preparation conditions and different data processing approaches.

